# Association between secondhand smoke and liver injury among US non-smoking adults: Mediation analysis of body mass index in the NHANES

**DOI:** 10.18332/tid/194489

**Published:** 2024-11-05

**Authors:** Mingcong Chen, Rongkun Luo, Zhao Lei, Feizhou Huang, Mingyi Zhao

**Affiliations:** 1 Department of Pediatrics, The Third Xiangya Hospital of Central South University, Changsha, China; 2 Department of Hepatopancreatobiliary Surgery, The Third Xiangya Hospital of Central South University, Changsha, China

**Keywords:** secondhand smoke, liver injury, BMI, mediation analyses, serum cotinine

## Abstract

**INTRODUCTION:**

Liver injury is a primary factor in the pathogenesis of most liver diseases, which can lead to liver failure. Secondhand smoke (SHS) is a serious public problem. This research explored the correlation between SHS and the indicators of liver injury.

**METHODS:**

This cross-sectional study was based on the National Health and Nutrition Examination Survey (NHANES) 2011–2016. The relationship between SHS and indicators of liver injury was explored by the weighted linear regression model and smooth curve fitting. The weighted threshold saturation effect model tested the relationship and inflection point between them. Mediation analyses were used to explore whether body mass index (BMI) mediates the correlation between SHS and liver injury indicators.

**RESULTS:**

Our cross-sectional study included 3811 non-smoking participants (aged 20–80 years). The full covariate adjustment model (β= -0.05; 95% CI: -0.08 – -0.02) showed a significant and negative correlation between log cotinine and albumin (ALB). Compared to the unexposed group, the ALB, and total protein (TP) were decreased by 0.16 g/dL, 0.26 g/dL in the heavy exposure group [ALB: -0.16 (-0.26 – -0.05), TP: -0.26 (-0.38 – -0.13)], respectively. Smoothed curve fitting revealed a nonlinear relationship between log cotinine and fibrosis-4 index (FIB-4 score), with the inflection point of log cotinine at -1.72. When log cotinine was < -1.72, the log cotinine significantly and positively correlated with the FIB-4 score (β=0.27; 95% CI: 0.06–0.49). BMI partially mediated the effect of SHS exposure on ALB or TP.

**CONCLUSIONS:**

SHS has harmful effects on the liver in never-smoking adults. BMI partially mediated the effect of SHS exposure on ALB or TP. More prospective and basic research in the future is necessary to focus on validating our results.

## INTRODUCTION

The liver is the largest anabolic organ in the human body that helps with digestion, hemostasis, and detoxification^1^. Liver injury is a major factor in the pathogenesis of most liver diseases, which can lead to liver failure, fibrosis, and cancer, posing a great threat to human liver health^2,3^. According to the Global Burden of Disease (GBD), >20000 people died of liver disease (liver failure, fibrosis, and cancer) worldwide in 2010, accounting for about 1% of all deaths^4^. According to the etiology, liver injury is divided into exogenous substances (drug-induced liver injury, etc.) and damage caused by disease and external stimulation (viral hepatitis, etc.)^5^. In addition, a large number of studies have shown that lifestyle can lead to liver injury^6^. Tobacco exposure, as one of the modifiable lifestyles, plays an important role in liver function damage.

Tobacco exposure includes active smoking and passive smoking. Studies have shown that the toxicity of sidestream smoke is 2–6 times that of mainstream smoke, that is, exposure to secondhand smoke (SHS) is more toxic than active smoking^7^. Among non-smokers, participants who were exposed to SHS had a 35% increased risk of heart failure compared to participants who were not exposed to SHS^8^. According to the World Health Organization survey, about 1.2 million non-smokers die from SHS every year^9^. At the same time, SHS is also one of the risk factors for cancer in non-smokers^10^. Lin et al.^11^ found that SHS was associated with non-alcoholic fatty liver disease (NAFLD) in children. Bhatta et al.^12^ found that SHS is closely related to the occurrence of liver cancer.

However, there is a lack of standardized and large-scale epidemiological studies in the current study to quantitatively study the relationship between SHS and liver injury. Therefore, we intend to explore the correlation between SHS exposure and liver injury in adults who never smoke, based on the National Health and Nutrition Examination Survey (NHANES) 2011–2016 data, which may contribute to the potential mechanism between SHS and liver injury.

## METHODS

### Study population and design

NHANES is a multi-stage, complex, and modified sampling cross-sectional survey that collects nutritional status and health-related information of adults and children in the United States. Since 1999, NHANES has updated its data for two consecutive years as a cycle. These data include physical examination data, demographic data, laboratory data, and dietary data. NHANES has received ethical approval from the Institutional Review Board of the Centers for Disease Control and Prevention. Participants signed an informed consent form.

**Figure 1:**
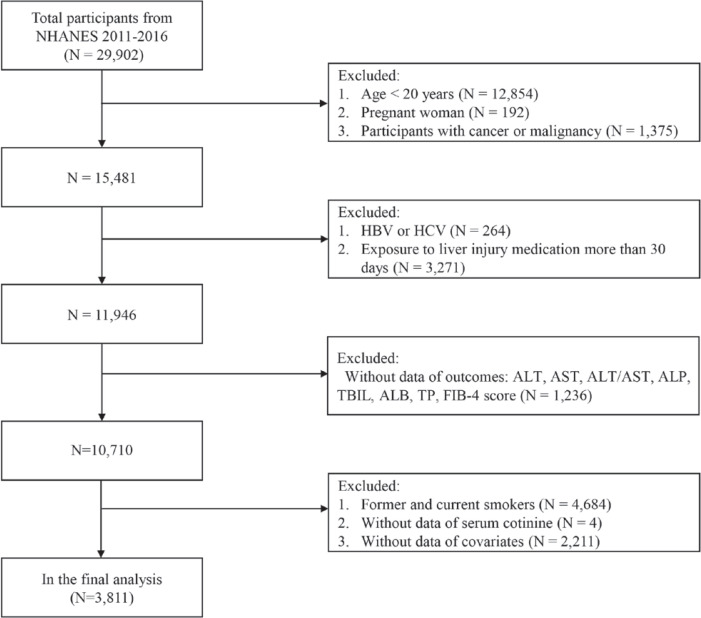
Flowchart of the study participants in the NHANES 2011–2016 (N=3811)

The present study derived data from NHANES 2011–2016. Among the 29902 subjects, we finally obtained 3811 available subjects based on the inclusion and exclusion criteria (Figure 1). The exclusion criteria were: 1) aged ≤20 years, 2) pregnant woman, 3) participants with cancer or malignancy, 4) participants with hepatitis B (positive HBsAg) or hepatitis C (positive HCV RNA), 5) taking drugs related to the liver injury for >30 days^13^, 6) without data on outcomes (the indicators of liver function/liver injury), 7) former and current smokers, and 8) incomplete serum cotinine and covariables.

### The indicators of exposures and outcomes

Serum cotinine is a tobacco-specific biomarker. According to the questionnaire and serum cotinine, non-smokers were defined as never using more than 100 cigarettes in their lifetime, not using nicotine-containing products in the last 5 days, and serum cotinine ≤10 ng/mL. Former smokers had smoked more than 100 cigarettes in their lifetime, not smoking now, and had not used nicotine-containing products in the past 5 days, and serum cotinine ≤10 ng/mL. Current smokers had smoked more than 100 cigarettes in their lifetime and currently smoking, or had used nicotine-containing products in the last 5 days, or had a serum cotinine >10 ng/mL^14^. SHS exposure was divided into three groups based on cotinine levels: unexposed (serum cotinine <0.05 ng/mL), low exposure (0.05 ng/mL ≤ serum cotinine <1 ng/mL), and heavy exposure (1 ng/mL ≤ serum cotinine <10 ng/mL)^14^.

The outcome variables of this study were the markers of liver injury, including serum alanine aminotransferase (ALT), aspartate aminotransferase (AST), AST/ALT, albumin (ALB), total protein (TP), total bilirubin (TBIL), alkaline phosphatase (ALP), and fibrosis-4 index (FIB-4)^15^. FIB-4 score is defined^16^ as:

Age (years) × AST (U/L)/[platelet (10^9^/L) × ALT (U/L)]^1/2^.

Serum samples were stored at -20^o^C and transported to the National Center for Environmental Health for testing. Serum cotinine was measured by isotope dilution-high performance liquid chromatography/atmospheric pressure chemical ionization tandem mass spectrometry (ID HPLC-APCI MS/MS). Serum cotinine levels below the detection limit were replaced by the detection limit/√2.

### Covariables

Covariables included education level, sex, age, race, marital status, physical activity category, hyperlipidemia, alcohol drinking, diabetes, hypertension, cardiovascular disease (CVD), family income-to-poverty ratio (PIR), and stroke.

PIR was an indicator representing the economic situation of the family, which was divided into three groups: poor (PIR <1), near poor (1≤ PIR <3), and non-poor (PIR ≥3)^17^. The participant blood pressure was taken after 5 minutes in a quiet sitting position. Hypertension was defined as systolic blood pressure ≥130 mmHg or diastolic blood pressure ≥85 mmHg, or taking antihypertensive drugs (self-reported questionnaire)^18^. Diabetes was defined as self-reported taking hypoglycemic drugs or insulin, or glycohemoglobin ≥6.5% or fasting blood glucose ≥126 mg/dL^19^. Physical activity was substituted by the metabolic equivalent of task (MET): below, <600 min/week; meet, 600 ≤ MET <1199 min/week; exceed, ≥1200 min/week^20^. Hyperlipidemia was defined as follows: 1) total cholesterol ≥200 mg/dL or LDL ≥130 mg/dL; 2) Female: HDL <50 mg/dL, male: HDL <40 mg/dL; or 3) participants were diagnosed with hyperlipidemia through the questionnaire survey^19^.

### Statistical analysis

This study used statistical software R (version 4.2.1) and EmpowerStats (version 2.0) for statistical analysis. Median (range) and a linear regression model described the continuous variables. The chi-squared test described categorial variables. All analyses were weighted appropriately. The distribution of serum cotinine was skewed, so to reduce the error, we log-transformed serum cotinine to make it normally distributed.

The relationship between SHS and liver injury indicators was explored through multiple linear regression. At the same time, the smooth curve fitting was employed to uncover the nonlinear relationship between SHS and liver injury indicators, and the threshold saturation effect model was used to test the relationship and inflection point between them. Finally, the same methods were used for subgroup analysis of sex, age, and race. The p for interaction was used to test whether there are differences between different subgroups. A p<0.05 (two-sided) was deemed statistical significance.

### Mediation effect analysis

The mediating effect was used to analyze whether BMI mediates the relationship between SHS and liver injury indicators, which was analyzed by using the R *MEDIATION* package. Path C was the total effect (TE) of SHS (X) on the liver injury indicators (Y) (Supplementary file Figure S1). Path C’ was the effect of SHS (X) on the liver injury indicators (Y) after adjusting for the effect of BMI (M). Path A was the effect of SHS (X) on the BMI (M), and Path B was the effect of BMI (M) on the liver injury indicators (Y). The TE of SHS was divided into direct effect (DE, path C’) and indirect effect (IE, path A*B) on liver injury indicators. In addition to the significant association of SHS (X) with liver injury indicators (Y) (path C, TE), when SHS (X) was significantly related to BMI (M), and BMI (M) was significantly related to liver injury indicators, the mediating effect exists^21^. The proportion of IE to TE (path A*B/ path C) indicated the efficacy of the mediators.

## RESULTS

### Basic characteristics of study subjects

The data analysis process is shown in Figure 1, a total of 3811 subjects met the inclusion criteria. The mean age of the research subjects was 42.07 ± 15.63 years, of which 44.37% were males. The baseline characteristics of subjects are displayed according to SHS exposure in Table 1. The participants with heavy SHS exposure were associated with lower age, ALB, FIB-4 score, and TP. They were more likely to be Mexican American, and other races/ethnicity, with less than a high school degree, married or living with a partner, non-poor, and high physical activity.

**Table 1:** Characteristics of the study population from NHANES 2011–2016, according to SHS exposure group, weighted (N=3811)

*Characteristics*	*Unexposed Median (range)*	*Low exposure Median (range)*	*Heavy exposure Median (range)*	*p*
Age (years)	41.50 (40.60–42.41)	35.41 (30.68–40.13)	31.83 (28.77–34.90)	<0.001
Platelet count (1000 cells/uL)	239.50 (236.79–242.21)	236.17 (223.73–248.61)	235.07 (212.19–257.96)	0.62
ALB (g/dL)	4.37 (4.35–4.39)	4.37 (4.25–4.48)	4.23 (4.14–4.32)	0.01
ALT (U/L)	24.72 (24.20–25.25)	23.29 (20.18–26.39)	25.32 (20.06–30.58)	0.56
AST/ALT	1.12 (1.10–1.14)	1.12 (1.02–1.21)	1.13 (1.00–1.26)	0.99
AST (U/L)	25.05 (24.56–25.54)	23.78 (21.81–25.76)	24.64 (21.89–27.38)	0.48
FIB-4 score	0.99 (0.93–1.04)	0.81 (0.66–0.95)	0.75 (0.63–0.88)	0.001
ALP (U/L)	63.20 (62.20–64.20)	64.01 (58.08–69.95)	91.09 (40.74–141.44)	0.55
TBIL (mg/dL)	0.68 (0.66–0.70)	0.67 (0.56–0.77)	0.65 (0.52–0.78)	0.86
TP (g/dL)	7.14 (7.11–7.16)	7.24 (7.12–7.37)	7.04 (6.91–7.17)	0.04
BMI (kg/m^2^)	28.46 (28.11–28.82)	29.38 (27.02–31.75)	31.89 (28.73–35.05)	0.098
**Sex,** %				0.36
Male	46.20 (44.50–47.90)	45.72 (30.62–61.65)	60.82 (35.97–81.09)	
Female	53.80 (52.10–55.50)	54.28 (38.35–69.38)	39.18 (18.91–64.03)	
**Race,** %				<0.001
Non-Hispanic White	63.96 (58.72–68.88)	57.97 (36.57–76.74)	29.71 (10.03–61.58)	
Non-Hispanic Black	9.85 (7.89–12.24)	27.97 (13.75–48.60)	27.14 (13.52–47.01)	
Mexican American	10.17 (7.60–13.48)	5.82 (2.17–14.71)	16.87 (4.72–45.39)	
Other race/ethnicity	16.02 (14.01–18.26)	8.24 (3.40–18.66)	26.28 (11.43–49.62)	
**Education level,** %				<0.001
Lower than high school	7.98 (6.41–9.90)	17.42 (6.94–37.38)	32.37 (13.51–59.47)	
High school	14.66 (12.58–17.02)	35.49 (20.95–53.32)	19.60 (9.26–36.79)	
Higher than high school	77.36 (73.79–80.57)	47.09 (35.34–59.18)	48.03 (27.93–68.78)	
**Marital status,** %				0.008
Never married	22.53 (19.79–25.54)	39.80 (23.06–59.33)	33.81 (17.77–54.70)	
Married or living with a partner	66.05 (63.14–68.85)	44.75 (28.70–61.98)	62.19 (42.02–78.87)	
Widowed, divorced, or separated	11.41 (10.14–12.81)	15.45 (6.41–32.74)	4.00 (0.72–19.36)	
**PIR,** %				<0.001
Poor	11.94 (9.77–14.52)	22.99 (13.02–37.32)	33.51 (17.42–54.63)	
Near poor	32.66 (29.41–36.10)	41.70 (30.22–54.14)	48.41 (29.30–67.99)	
Non-poor	55.40 (51.11–59.60)	35.31 (18.40–56.92)	18.08 (8.07–35.70)	
**Hypertension,** %				0.70
No	66.06 (64.04–68.02)	61.64 (48.33–73.40)	62.81 (41.91–79.81)	
Yes	33.94 (31.98–35.96)	38.36 (26.60–51.67)	37.19 (20.19–58.09)	
**Physical activity category,** %				0.008
Below	46.60 (44.08–49.14)	32.10 (17.86–50.68)	37.49 (19.44–59.84)	
Meet	24.63 (22.83–26.52)	22.82 (10.28–43.28)	10.45 (4.21–23.64)	
Exceed	28.77 (26.74–30.89)	45.09 (31.05–59.96)	52.06 (31.21–72.22)	
**Hypermedia,** %				0.49
No	36.71 (34.12–39.38)	41.64 (27.31–57.54)	44.06 (28.67–60.69)	
Yes	63.29 (60.62–65.88)	58.36 (42.46–72.69)	55.94 (39.31–71.33)	
**Alcohol drinking,** %				0.08
Never	16.32 (12.83–20.53)	12.05 (3.84–31.96)	16.19 (7.29–32.18)	
Former	8.93 (7.76–10.27)	9.89 (4.57–20.10)	2.41 (0.22–21.94)	
Mild	40.96 (37.40–44.62)	26.68 (15.35–42.22)	31.70 (13.31–58.38)	
Moderate	18.06 (16.35–19.91)	26.09 (11.61–48.70)	16.26 (6.42–35.46)	
Heavy	15.72 (13.79–17.87)	25.28 (12.76–43.90)	33.45 (17.15–54.97)	
**Diabetes,** %				0.56
No	93.14 (91.81–94.27)	96.61 (88.73–99.04)	94.76 (66.54–99.40)	
Yes	6.86 (5.73–8.19)	3.39 (0.96–11.27)	5.24 (0.60–33.46)	
**CVD,** %				0.23
No	98.70 (98.23–99.05)	100.00 (100.00–100.00)	94.76 (66.54–99.40)	
Yes	1.30 (0.95–1.77)	0.00 (0.00–0.00)	5.24 (0.60–33.46)	
**Stroke,** %				0.83
No	99.01 (98.54–99.33)	98.97 (90.96–99.89)	100.00 (100.00–100.00)	
Yes	0.99 (0.67–1.46)	1.03 (0.11–9.04)	0.00 (0.00–0.00)	

Continuous variables: the p-value was calculated by the weighted linear regression model. Categorical variables: the p-value was calculated by the weighted chi-squared test.

SHS exposure groups: unexposed (serum cotinine <0.05 ng/mL), low exposure (0.05 ng/mL ≤ serum cotinine <1 ng/mL), heavy exposure (1 ng/mL ≤ serum cotinine <10 ng/mL). ALB: albumin. ALT: alanine aminotransferase. AST: aspartate aminotransferase: FIB-4: fibrosis-4 index. ALP: alkaline phosphatase. TBIL: total bilirubin. TP: total protein. BMI: body mass index. PIR: family income-to-poverty ratio. CVD: cardiovascular disease.

### Effects of SHS exposure on liver injury indicators

To further discover the underlying correlation between SHS exposure and liver injury indicators, we utilized the multivariable linear regression analysis to organize the fully adjusted model (all the covariables were adjusted) depicted in Table 2. We found that the log cotinine was significant and negatively associated with ALB (β= -0.05; 95% CI: -0.08 – -0.02). After adjusting for all the covariables, we further divided SHS exposure into three groups based on serum cotinine levels, namely, unexposed, low exposure, and heavy exposure. Compared to the unexposed group, the ALB and TP were decreased by 0.16 g/dL, 0.26 g/dL in the heavy exposure group [ALB: -0.16 (-0.26 – -0.05), TP: -0.26 (-0.38 – -0.13)], respectively. And a significant linear trend was found (p<0.05).

**Table 2:** Association of log cotinine and SHS exposure group, and the indicators of liver function/injury, NHANES 2011–2016 (N=3811)

*Outcomes*	*Log cotinine (ng/mL)*	*Low exposure*	*Heavy exposure*	*p*
	*Adjusted β (95% CI)*	*Adjusted β (95% CI)*	*Adjusted β (95% CI)*	
ALT (U/L)	-0.48 (-1.90–0.95)	-1.46 (-4.02–1.1)	-1.80 (-6.55–2.95)	0.32
AST (U/L)	-0.31 (-1.21–0.59)	-1.77 (-4.12–0.57)	-1.08 (-3.99–1.83)	0.53
AST/ALT	0.01 (-0.02–0.03)	-0.02 (-0.09–0.05)	0.05 (-0.04–0.14)	0.21
ALP (U/L)	2.59 (-1.58–6.77)	-0.01 (-5.43–5.42)	26.67 (-24.76–78.10)	0.07
TBIL (mg/dL)	0.01 (-0.02–0.03)	0.011 (-0.09–0.11)	-0.02 (-0.16–0.13)	0.94
ALB (g/dL)	-0.05 (-0.08 – -0.02)**	0.01 (-0.11–0.13)	-0.16 (-0.26 – -0.05)**	0.02
TP (g/dL)	-0.02 (-0.06–0.01)	0.03 (-0.09–0.15)	-0.26 (-0.38 – -0.13)***	0.001
FIB-4 score	-0.001 (-0.05–0.05)	-0.01 (-0.10–0.08)	0.08 (-0.07–0.23)	0.57

Reference: unexposed. Adjusted β: adjusted for sex, age, race, education level, marital status, PIR, hypertension, physical activity category, hypermedia, alcohol drinking, diabetes, CVD, and stroke. SHS exposure groups: unexposed (serum cotinine <0.05 ng/mL), low exposure (0.05 ng/mL ≤ serum cotinine < 1 ng/mL), heavy exposure (1 ng/mL ≤ serum cotinine <10 ng/mL). SHS: secondhand smoke. ALB: albumin. ALT: alanine aminotransferase. AST: aspartate aminotransferase. FIB-4: fibrosis-4 index. ALP: alkaline phosphatase. TBIL: total bilirubin. TP: total protein. PIR: family income-to-poverty ratio. CVD: cardiovascular disease.

* p<0.05.

** p<0.01.

*** p<0.001.

Supplementary file Figure S2 reveals the correlation between log cotinine and liver injury indicators through smooth curve fitting after adjusting all the covariables. We found that there were curve relationships between log cotinine and AST, FIB-4 score and TP (Supplementary file Figures S2B, S2G, and S2H), with the inflection points of -1.49, -1.72, -0.55, and -0.38, respectively (Table 3). When log cotinine (ng/mL) was < -1.72, the log cotinine significantly and positively correlated with the FIB-4 score (β=0.27; 95% CI: 0.06–0.49). When the log cotinine was ≥ -0.38, there was a reduction of TP with increasing log cotinine (β= -0.15; 95% CI: -0.26 – -0.04).

**Table 3:** Threshold effect analysis of log-transformed serum cotinine (ng/mL) on the indicators of liver function/injury using the piecewise linear regression model, NHANES 2011–2016 (N=3811)

*Outcomes*	*Adjusted β (95% CI)*
**AST**	
Inflection point	-1.49
Log cotinine < -1.49	3.02 (-0.43–6.48)
Log cotinine ≥ -1.49	-1.27 (-3.05–0.51)
Log likelihood ratio p	0.065
**FIB-4 score**	
Inflection points	-1.72, -0.55
Log cotinine < -1.72	0.27 (0.06–0.49)*
-1.72 ≤ log cotinine < -0.55	-0.07 (-0.16–0.02)
Log cotinine ≥ -0.55	0.09 (-0.08–0.26)
Log likelihood ratio p	0.008
**TP (g/dL)**	
Inflection point	-0.38
Log cotinine < -0.38	-0.01 (-0.04–0.03)
Log cotinine ≥ -0.38	-0.15 (-0.26 – -0.04)**
Log likelihood ratio p	0.029

Analysis based on fully adjusted model assessing the non-linear association with the potential threshold effect between log-transformed serum cotinine and the indicators of liver function/liver injury. Log-likelihood ratio was used to compare the one-line linear regression model and the piecewise linear regression model. A p<0.05 indicates that the fitting effect between the piecewise linear regression models and the data is significantly better than that of the one-line linear regression model. Fully adjusted model: sex, age, race, education level, marital status, PIR, hypertension, physical activity category, hypermedia, alcohol drinking, diabetes, CVD, and stroke were adjusted. SHS: secondhand smoke. ALB: albumin. ALT: alanine aminotransferase.

AST: aspartate aminotransferase. FIB-4: fibrosis-4 index. ALP: alkaline phosphatase.

TBIL: total bilirubin. TP: total protein. PIR: family income-to-poverty ratio. CVD: cardiovascular disease.

* p<0.05.

** p<0.01.

Supplementary file Tables 1–3 reveal the correlation between log cotinine and liver injury indicators based on sex, age, and ethnic stratification. As shown in Supplementary file Table 1, the log cotinine was negatively related with ALB (β= -0.07; 95% CI: -0.11 – -0.04) in males. The analysis of Supplementary file Table 2, showed the negative relationship between log cotinine and ALB in non-Hispanic White, non-Hispanic Black, and other race/ethnicity. At the same time, we found that log cotinine was negatively associated with ALT in other races/ethnicities (β= -3.23; 95% CI: -5.60 – -0.47). As shown in Supplementary file Table 3, the log cotinine negatively correlated with ALB (β= -0.05; 95% CI: -0.08 – -0.02] in those aged 20–39 years. All the p>0.05.

### Roles of BMI in the relationships of SHS exposure with liver injury indicators

**Figure 2:**
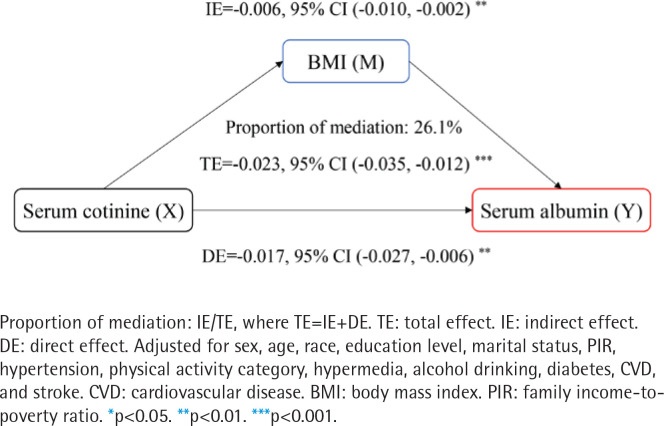
Mediation analysis of BMI on the interaction between log-transformed serum cotinine and albumin, NHANES 2011–2016 (N=3811)

**Figure 3:**
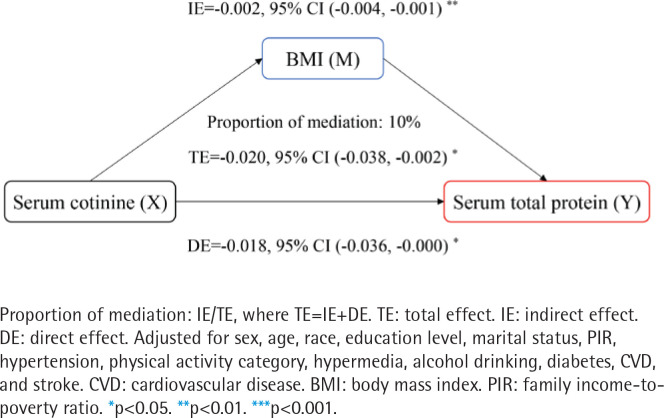
Mediation analysis of BMI on the interaction between log-transformed serum cotinine and total protein, NHANES 2011–2016 (N=3811)

We further performed whether BMI acts as a mediator in the relationships between SHS exposure and ALB, TP, or FIB-4 score (Figures 2 and 3, and Supplementary file Figure S3). As presented in Figure 2, the BMI effect contributed to 26.1% of the TE of log cotinine on the ALB (TE= -0.023; 95% CI: -0.035 – -0.012, IE= -0.006; 95% CI: -0.010 – -0.002). As presented in Figure 3, the BMI effect contributed to 10% of the TE of log cotinine on the TP (TE= -0.020; 95% CI: -0.038 – -0.002, IE= -0.002; 95% CI: -0.004 – -0.001). There was no significant mediating effect of the FIB-4 score (TE=0.007; 95% CI: -0.009–0.024). Moreover, we found that after adjusting for BMI, the relationship between SHS exposure and ALB or TP was weakened but significant (ALB: DE= -0.017; 95% CI: -0.027 – -0.006; TP: DE= -0.018; 95% CI: -0.036 – -0.000). Thus, BMI was considered to be a partial mediator in this relationship^22^.

## DISCUSSION

We explored the relationship between SHS exposure with liver injury indicators. In our cross-sectional study, based on never smoker participants aged >20 years from the NHANES database, we discovered that SHS exposure is inversely related to liver injury indicators (ALB and TP). When log cotinine (ng/mL) was < -1.72, the log cotinine significantly and positively correlated with the FIB-4 score by the piecewise linear regression approach. Also, we analyzed the relationships between SHS exposure and liver injury indicators from the aspects of age, sex, and ethnic stratification. Furthermore, BMI partially mediated the effect of SHS exposure on ALB or TP.

SHS contains a variety of chemicals and carcinogenic substances and is a public health problem, associated with various diseases in adults and children^23^. People who are exposed to SHS have an increased risk of liver cancer, lung cancer, stroke, childhood asthma, and other diseases^23^. SHS may aggravate oxidative stress damage and further induce accelerated brain aging^24^. The metal concentrations of mainstream and sidestream smoke inhaled by passive smokers are different^25^. Mansouri et al.^26^ found that women exposed to SHS had higher levels of toxic elements such as As, Cd, Hg, and Pb in breast milk than women not exposed to SHS. A study found that the levels of liver and kidney-related damage indicators increased in rats exposed to tobacco^27^. This is consistent with the results of this study that SHS is closely related to liver injury. This may be related to the production of free radicals by the body. After SHS enters the body, nicotine is oxidized to cotinine and increases intracellular production of reactive oxygen species by inducing mitochondrial dysfunction^28^. When the concentration of reactive oxygen species exceeds the body’s antioxidant capacity, the body’s oxidation/antioxidant capacity is disordered, which in turn aggravates liver damage^27^.

The mediating analysis of this study showed that the effect of SHS on liver injury was partially mediated by BMI. This study showed that SHS was positively correlated with BMI. One animal study found that SHS exposure induced up-regulation of cholesterol synthesis-related genes^29^ and led to an increased risk of obesity^30^. In addition, SHS can further induce liver fatty acid synthesis by regulating AMP-activated protein kinase (AMPK) and sterol regulatory element binding protein-1c (SREBP-1c)^31,32^.

Obesity can lead to excessive production of type I collagen and accumulation of extracellular matrix (ECM) in the liver, which further forms scar tissue and eventually leads to liver injury^33,34^.

### Strengths and limitations

There are some strengths in this study. Firstly, this is the first study to investigate the relationship between SHS exposure and liver injury indicators in people in USA based on a large sample. Secondly, this study uses the appropriate weights to ensure that the results of this study are representative. Thirdly, the definition of SHS in this study is derived from self-reported and serum cotinine levels, which further reduces subjectivity.

Our study also has limitations. Firstly, this is a cross-sectional study that can only conclude there is a correlation between SHS and liver injury indicators rather than a causal relationship. More prospective and basic research in the future is necessary to focus on validating our results. Secondly, the subjects were only USA residents and do not represent the populations of other countries. The results need to be further verified with external data. Thirdly, the level of cotinine in the serum has a certain half-life, which can only indicate recent exposure. Finally, in reality, numerous covariables can influence SHS and liver injury indicators. It is challenging to include all these covariates.

## CONCLUSIONS

SHS has harmful effects on the liver in never smoker adults. BMI partially mediated the effect of SHS exposure on ALB or TP. This indicates that SHS is positively correlated with liver injury, and avoiding daily exposure to SHS may help prevent liver injury. More prospective and basic research in the future is necessary to focus on validating our results.

## Supplementary Material



## Data Availability

The NHANES datasets can be found online (https://wwwn.cdc.gov/nchs/nhanes/). The data supporting this research are available from the following source: https://www.cdc.gov/nchs/nhanes/index.htm
