# Supplementary material for: Association between secondhand smoke and liver injury among US non-smoking adults: Mediation analysis of body mass index in the NHANES
Source: Tob Induc Dis. 2024 Nov 5;22:10.18332/tid/194489. doi: 10.18332/tid/194489 (PMC11536516; doi:10.18332/tid/194489)
Supplement: Supplementary file 1 [file TID-22-173-s1.pdf]

Supplementary Table S1. Association between log-transformed serum cotinine and liver function parameters stratified by sex, NHANES 2011-2016 (N=3811)

Outcomes	No. of participants	log cotinine, ng/mL	*P* for interaction
FIB- 4 score			
Male	1691	0.04 (-0.05, 0.13)	
Female	2120	-0.03 (-0.09, 0.03)	0.32
ALB			
Male	1691	-0.07 (-0.11, -0.04) ***	
Female	2120	-0.02 (-0.06, 0.01)	0.88
ALT			
Male	1691	-0.53 (-2.77, 1.70)	
Female	2120	-0.69 (-1.87, 0.48)	0.06
AST			
Male	1691	-0.19 (-1.60, 1.21)	
Female	2120	-0.63 (-1.78, 0.51)	0.009
AST/ALT			
Male	1691	0.01 (-0.03, 0.04)	
Female	2120	0.01 (-0.03, 0.04)	0.69
AKP			
Male	1691	4.82 (-2.79, 12.44)	
Female	2120	0.31 (-1.73, 2.34)	0.14
TBIL			
Male	1691	0.02 (-0.02, 0.06)	
Female	2120	-0.00 (-0.00, 0.00)	0.41
TP			
Male	1691	-0.03 (-0.08, 0.03)	
Female	2120	-0.02 (-0.07, 0.02)	0.31

Note: Data are β coefficients (95% CIs) *P*-values.

*** P-value <0.001.

Age, race, education level, marital status, PIR, hypertension, physical activity category, hypermedia, alcohol drinking, diabetes, CVD, and stroke were adjusted.

Abbreviations: NHANES, National Health and Nutrition Examination Survey; ALB, albumin; ALT, alanine aminotransferase; AST, aspartate aminotransferase; FIB-4, fibrosis-4 index; ALP, alkaline phosphatase; TBIL, total bilirubin; TP, total protein; PIR, family income-to-poverty ratio; CVD, cardiovascular disease.

Supplementary Table S2. Association between log-transformed serum cotinine and liver function parameters stratified by race, NHANES 2011-2016 (N=3811)

Outcomes	No. of participants	log cotinine, ng/mL	*P* for interaction
FIB- 4 score			
Non-Hispanic white	1294	-0.01 (-0.10, 0.07)	
Non-Hispanic black	771	0.00 (-0.06, 0.07)	
Mexican American	599	0.02 (-0.05, 0.09)	
Other race/ethnicity	1147	0.02 (-0.02, 0.06)	0.3
ALB			
Non-Hispanic white	1294	-0.05 (-0.10, -0.01) *	
Non-Hispanic black	771	-0.05 (-0.07, -0.02) **	
Mexican American	599	-0.00 (-0.05, 0.05)	
Other race/ethnicity	1147	-0.05 (-0.10, -0.01) *	0.06
ALT			
Non-Hispanic white	1294	0.23 (-1.86, 2.33)	
Non-Hispanic black	771	-1.83 (-4.15, 0.49)	
Mexican American	599	2.62 (-2.22, 7.46)	
Other race/ethnicity	1147	-3.23 (-5.60, -0.47) *	0.1
AST			
Non-Hispanic white	1294	-0.50 (-1.73, 0.72)	
Non-Hispanic black	771	-0.77 (-2.94, 1.39)	
Mexican American	599	2.32 (-2.41, 7.05)	
Other race/ethnicity	1147	-1.23 (-3.03, 0.57)	0.51
AST/ALT			
Non-Hispanic white	1294	-0.02 (-0.05, 0.02)	
Non-Hispanic black	771	0.02 (-0.02, 0.07)	
Mexican American	599	0.00 (-0.05, 0.05)	
Other race/ethnicity	1147	0.03 (-0.01, 0.07)	0.44
AKP			
Non-Hispanic white	1294	4.53 (-1.88, 10.94)	
Non-Hispanic black	771	0.22 (-3.02, 3.47)	
Mexican American	599	0.40 (-2.72, 3.52)	
Other race/ethnicity	1147	-0.00 (-2.38, 2.37)	0.51
TBIL			
Non-Hispanic white	1294	0.02 (-0.01, 0.05)	
Non-Hispanic black	771	-0.03 (-0.07, 0.01)	
Mexican American	599	0.02 (-0.04, 0.08)	
Other race/ethnicity	1147	-0.02 (-0.08, 0.03)	0.19
TP			
Non-Hispanic white	1294	-0.01 (-0.07, 0.04)	
Non-Hispanic black	771	-0.06 (-0.12, -0.00) *	
Mexican American	599	0.02 (-0.05, 0.09)	
Other race/ethnicity	1147	-0.05 (-0.11, 0.02)	0.15

Note: Data are β coefficients (95% CIs) P-values.

* P-value <0.05; ** P-value <0.01.
Age, sex, education level, marital status, PIR, hypertension, physical activity category, hypermedia, alcohol drinking, diabetes, CVD, and stroke were adjusted.
Abbreviations: NHANES, National Health and Nutrition Examination Survey; ALB, albumin; ALT, alanine aminotransferase; AST, aspartate aminotransferase; FIB-4, fibrosis-4 index; ALP, alkaline phosphatase; TBIL, total bilirubin; TP, total protein; PIR, family income-to-poverty ratio; CVD, cardiovascular disease.

Supplementary Table S3. Association between log-transformed serum cotinine and liver function parameters stratified by age, NHANES 2011-2016 (N=3811)

Outcomes	No. of participants	log cotinine, ng/mL	*P* for interaction
FIB- 4 score			
20-39 years	1855	-0.00 (-0.03, 0.02)	
40-59 years	1326	-0.07 (-0.15, 0.02)	
60-80 years	630	0.03 (-0.24, 0.30)	0.39
ALB			
20-39 years	1855	-0.05 (-0.08, -0.02) **	
40-59 years	1326	-0.04 (-0.11, 0.03)	
60-80 years	630	-0.03 (-0.09, 0.02)	0.25
ALT			
20-39 years	1855	-0.46 (-2.27, 1.34)	
40-59 years	1326	0.13 (-2.32, 2.58)	
60-80 years	630	-1.55 (-3.61, 0.51)	0.6
AST			
20-39 years	1855	-0.13 (-1.28, 1.03)	
40-59 years	1326	0.17 (-1.76, 2.10)	
60-80 years	630	-2.16 (-4.11, -0.20) *	0.11
AST/ALT			
20-39 years	1855	0.01 (-0.02, 0.04)	
40-59 years	1326	-0.01 (-0.06, 0.04)	
60-80 years	630	-0.01 (-0.07, 0.04)	0.37
AKP			
20-39 years	1855	3.02 (-2.51, 8.54)	
40-59 years	1326	2.75 (-0.91, 6.40)	
60-80 years	630	-0.59 (-4.10, 2.91)	0.31
TBIL			
20-39 years	1855	-0.01 (-0.04, 0.03)	
40-59 years	1326	0.038 (-0.01, 0.09)	
60-80 years	630	0.012 (-0.04, 0.07)	0.19
TP			
20-39 years	1855	-0.01 (-0.05, 0.03)	
40-59 years	1326	-0.02 (-0.11, 0.06)	
60-80 years	630	-0.07 (-0.17, 0.03)	0.73

Note: Data are β coefficients (95% CIs) P-values.

* P-value <0.05; ** P-value <0.01.

Race, sex, education level, marital status, PIR, hypertension, physical activity category, hypermedia, alcohol drinking, diabetes, CVD, and stroke were adjusted.

Abbreviations: NHANES, National Health and Nutrition Examination Survey; ALB, albumin; ALT, alanine aminotransferase; AST, aspartate aminotransferase; FIB-4, fibrosis-4 index; ALP, alkaline phosphatase; TBIL, total bilirubin; TP, total protein; PIR, family income-to-poverty ratio; CVD, cardiovascular disease.

Supplementary Figure S1. Schematic diagram of mediation pathway analysis

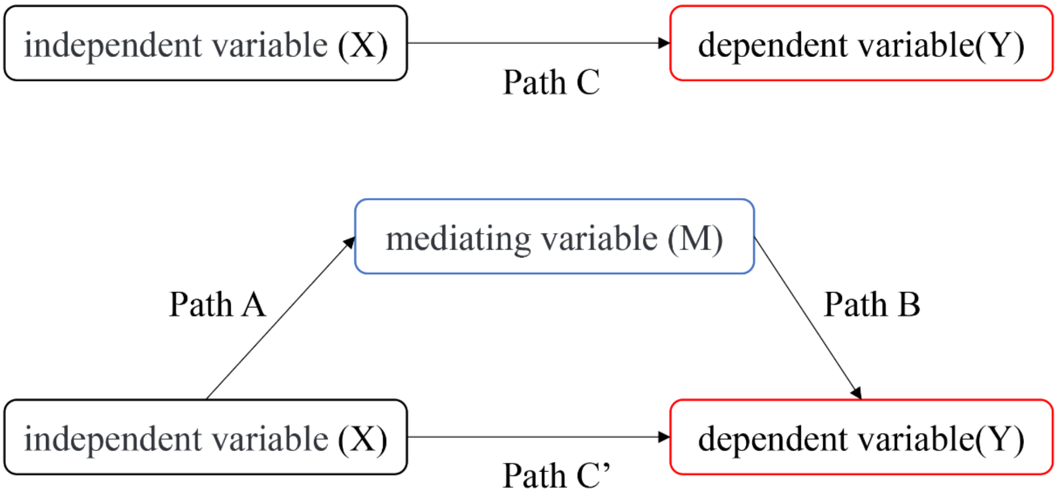

Path C: the TE of X (independent) on Y (dependent). Path C': the effect of X on Y after adjusting for the effect of M (mediating variable). Path A: the effect of X on M. Path B: the effect of M on the Y.

Abbreviations: TE, total effect.

Supplementary Figure S2. The association between log-transformed serum cotinine and the indicators of liver function/liver injury through smooth curve fitting, NHANES 2011-2016 (N=3811)

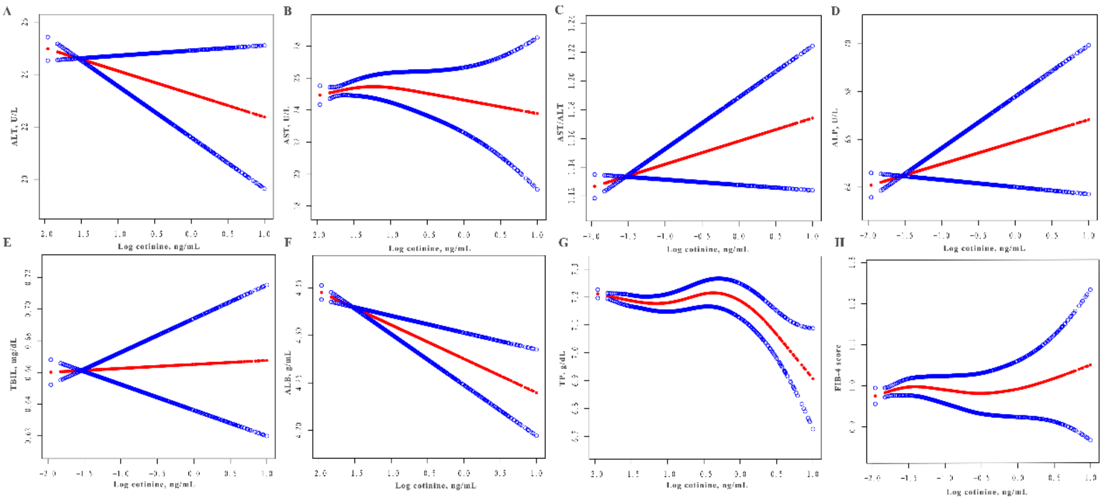

(A) The association between log cotinine and ALT; (B) The association between log cotinine and AST; (C) The association between log cotinine and AST/ALT; (D) The association between log cotinine and ALP; (E) The association between log cotinine and TBIL; (F) The association between log cotinine and ALB; (G) The association between log cotinine and TP; (H) The association between log cotinine and FIB-4 score.

The red line represents the estimated values. The blue line indicates the 95% confidence interval for the fit. They were adjusted for sex, age, race, education level, marital status, PIR, hypertension, physical activity category, hypermedia, alcohol drinking, diabetes, CVD, and stroke.
Abbreviations: ALB, albumin; ALT, alanine aminotransferase; AST, aspartate aminotransferase; FIB-4, fibrosis-4 index; ALP, alkaline phosphatase; TBIL, total bilirubin; TP, total protein; PIR, family income-to-poverty ratio; CVD, cardiovascular disease.

Supplementary Figure S3. Mediation analysis of BMI on the interaction between log-transformed serum cotinine and total FIB-4 score, NHANES 2011-2016 (N=3811)

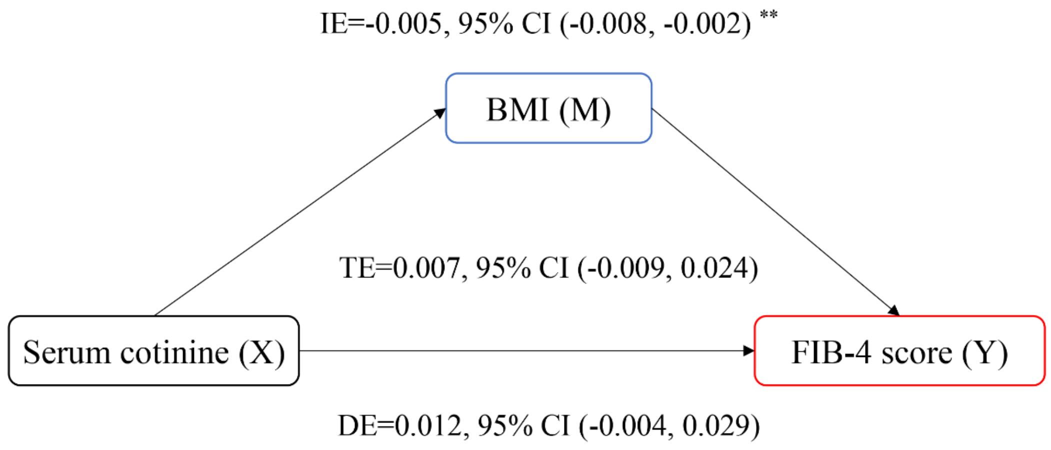

Note: * P-value <0.05; ** P-value <0.01; *** P-value <0.001.
Proportion of mediation=IE/TE, TE=IE+DE.
They were adjusted for sex, age, race, education level, marital status, PIR, hypertension, physical activity category, hypermedia, alcohol drinking, diabetes, CVD, and stroke.
Abbreviations: TE, total effect; IE, indirect effect; DE, direct effect; BMI, body mass index; PIR, family income-to-poverty ratio; CVD, cardiovascular disease.

© 2024 Chen M. et al.
